# Contribution of Secondary Metabolites to the Gastroprotective Effect of Aqueous Extract of *Ximenia americana* L. (Olacaceae) Stem Bark in Rats

**DOI:** 10.3390/molecules23010112

**Published:** 2018-01-09

**Authors:** Ticiana Parente Aragão, Lady Dayane Kalline Travassos dos Prazeres, Samara Alves Brito, Pedro José Rolim Neto, Larissa Araújo Rolim, Jackson Roberto Guedes da Silva Almeida, Germana Freire Rocha Caldas, Almir Gonçalves Wanderley

**Affiliations:** 1 Department of Pharmaceutical Sciences, Federal University of Pernambuco, Recife 50740-521, PE, Brazil; ticianaragao@yahoo.com.br (T.P.A.); kallinetravassos@gmail.com (L.D.K.T.d.P.); samaralvesbritobrito19@gmail.com (S.A.B.); 2 Department of Nutrition, University of Pernambuco, Petrolina 56328-903, PE, Brazil; 3 Laboratory of Medication Technology, Federal University of Pernambuco, Recife 50740-521, PE, Brazil; rolim.pedro@gmail.com; 4 Central of Analysis of Drugs, Medicines and Food, Federal University of San Francisco Valley, Petrolina 56304-205, PE, Brazil; larissa.rolim@univasf.edu.br; 5 Center for Studies and Research of Medicinal Plants, Federal University of San Francisco Valley, Petrolina 56304-205, PE, Brazil; jackson.guedes@univasf.edu.br; 6 Graduate Program in Health Sciences, Biological and Health Sciences Center, Federal University of Maranhão, São Luís 65080-805, MA, Brazil; germanafreire@yahoo.com.br; 7 Department of Physiology and Pharmacology, Federal University of Pernambuco, Recife 50670-901, PE, Brazil

**Keywords:** *Ximenia*, Olacaceae, flavonoids, gastric lesion

## Abstract

*Ximenia americana* L. (Olacaceae) is used in ethnomedicine as cicatrizant and for the treatment of gastric disorders. This study identified the chemical constituents of the aqueous extract of *X. americana* (XaAE) and evaluated its antiulcerogenic activity. After lyophilization, XaAE was analyzed by liquid chromatography-mass spectrometry (LC-MS) and its antiulcerogenic effect was evaluated in acute gastric lesions induced by ethanol, acidified ethanol, and indomethacin. Antisecretory action, mucus production and the participation of sulfhydryl groups (–SH) and nitric oxide (NO) were also investigated. The chromatographic analysis identified procyanidins B and C and catechin/epicatechin as major compounds. Oral administration of XaAE (100, 200 and 400 mg/kg) inhibited the gastric lesions induced by ethanol (76.1%, 77.5% and 100%, respectively), acidified ethanol (44.9%, 80.6% and 94.9%, respectively) and indomethacin (56.4%, 52.7% and 64.9%, respectively). XaAE reduced gastric contents and acidity (51.4% and 67.7%, respectively) but did not alter the production of gastric mucus. The reduction of the -SH and NO groups promoted by *N*-ethylmaleimide (NEM) and *N*ω-nitro-l-arginine-methyl-ester (L-NAME) respectively, reduced the gastroprotective effect of XaAE. In conclusion, XaAE has gastroprotective activity mediated in part by -SH, NO and antisecretory activity. This antiulcer action was initially correlated to its major constituents, procyanidins B and C and catechin/epicatechin.

## 1. Introduction

Peptic ulcer disease is a chronic, multifactorial disease characterized by lesions that affect the mucosa of the esophagus, stomach and/or duodenum, and may extend to the muscular layer of the mucosa [1]. Lesions occur due to an imbalance between the defensive factors (mucus, bicarbonate, mucosal blood flow, endogenous prostaglandins) and aggressors (acid secretion, alcohol, nonsteroidal anti-inflammatory drugs, *Helicobacter pylori* and reactive oxygen species) of the gastric mucosa [2].

These lesions affect about four million people worldwide, more often men than women, with duodenal ulcer more prevalent among young people and gastric ulcer among the elderly [1,3]. Currently, treatment options for peptic ulcer are based on the use of antacids, cytoprotective agents, muscarinic antagonists, H_2_ antihistamines, proton pump inhibitors, and more recently the use of antimicrobials for the treatment of *Helicobacter pylori* infection [4,5]. These drugs have led to a reduction in the incidence and prevalence of peptic ulcers. However, the side effects of chronic treatment with these drugs and the increased resistance of *H. pylori* to antibiotics indicate the need to search new substances that may help in the treatment of this disease.

The inhibition of gastric acid secretion with anti-secretory drugs such as proton-pump inhibitors or H_2_-receptor antagonists has been a viable therapeutic option in the treatment of gastric diseases, despite the recurrence and/or adverse effects [6]. In this context, we used pantoprazole or ranitidine as standard drugs in the experimental models. In addition, carbenoxolone was also used as a standard drug in some experimental models considering its potent cytoprotective gastric action [7].

*X. americana* L. (Olacaceae), commonly known as prickly plum, was first described and named in 1606, in Australia, by the Spanish naturalist Francisco Ximenes [8]. It is a tropical, uncultivated and uncommon natural cosmopolitan plant, occurring mainly in Africa and South America, including on the coastal plateaus in the Northeast Brazil [9].

In ethnomedicine, *X. americana* stem bark is used as an astringent and healing agent, and is also indicated for the treatment of wounds, skin and mucosal ulcerations, gastritis and stomach discomfort [9]. Some studies have explored its ulcer healing, anti-inflammatory [10,11], analgesic [12], antioxidant [13,14], antimicrobial [15], antihelmintic [16] and anticancer [17] action.

The lack of literature support for the action of *X. americana* L. stem bark on the treatment of gastric problems, whose use is based on ethnopharmacological information, motivated us to explore the possible antiulcerogenic action of this species in acute preclinical models of gastric lesions.

## 2. Results

### 2.1. Phytochemical Profile

The analysis revealed the presence of high levels of phenolic compounds, lignans, monoterpenes, sesquiterpenes, diterpenes, naphthoquinones, triterpenes and steroids, and smaller quantities of alkaloids, saponins and hydrolyzable tannins (Table 1).

### 2.2. High Performance Liquid Chromatography (HPLC-DAD-MS/MS)

**Figure 1:**
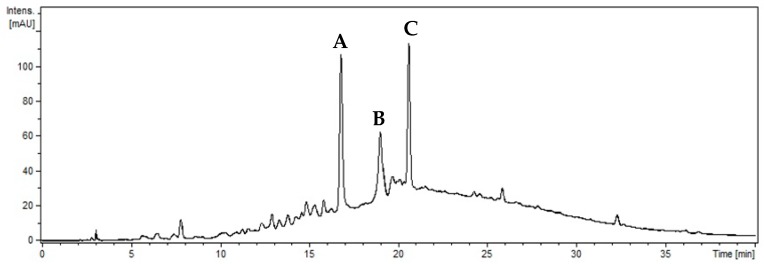
Chromatogram of *X. americana* extract at 270 nm. (**A**) Procyanidin B; (**B**) Procyanidin C; (**C**) Catechin/epicatechin.

**Figure 2:**
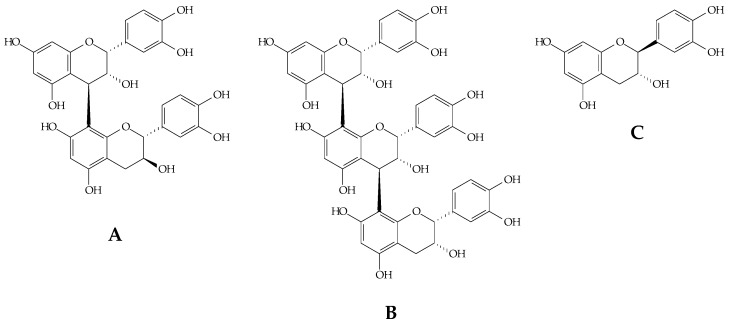
Structures of the major constituents of *X. americana* L. stem bark (**A**) Procyanidin B; (**B**) Procyanidin C; (**C**) Catechin/epicatechin.

The chromatographic analysis of the aqueous extract of *X. americana* suggested the presence of procyanidin B, procyanidin C and catechin/epicatechin compounds for the similarity between ultraviolet absorption spectrum, mass/charge ratio and fragmentation (MS^2^) as described in Table 2. The precursor ions and fragmentation spectra of the major constituents in the *m*/*z* extract were 577.16 (A), 866.19 (B) and 289.03 (C) and are shown in Figure 1 and Figure 2 to prove the identity of procyanidins B, procyanidins C, and catechin/epicatechin, respectively.

The UV chromatogram at 270 nm, Figure 1, shows that the major constituents in the *X. americana* extract are procyanidins. Although it was not possible to identify them, they are procyanidins B1, B2, B3 or B4 because they have the same fragmentation profile as described by other authors [18].

### 2.3. Acute Toxicity

Oral administration of 2000 mg/kg of XaAE did not induce death nor produced visible signs of behavioral changes or toxicity in the treated mice during the 14 days of observation. There were no significant differences in water intake (9.24 ± 0.35 vs. 8.66 ± 0.29 mL/day/animal), food consumption (5.32 ± 0.13 vs. 5.57 ± 0.09 g/day/animal), and body weight (36.67 ± 0.27 vs. 36.33 ± 0.99 g) between the control and treated group, respectively.

### 2.4. Experimental Protocols

#### 2.4.1. Induction of Gastric Lesions by Absolute Ethanol

**Figure 3:**
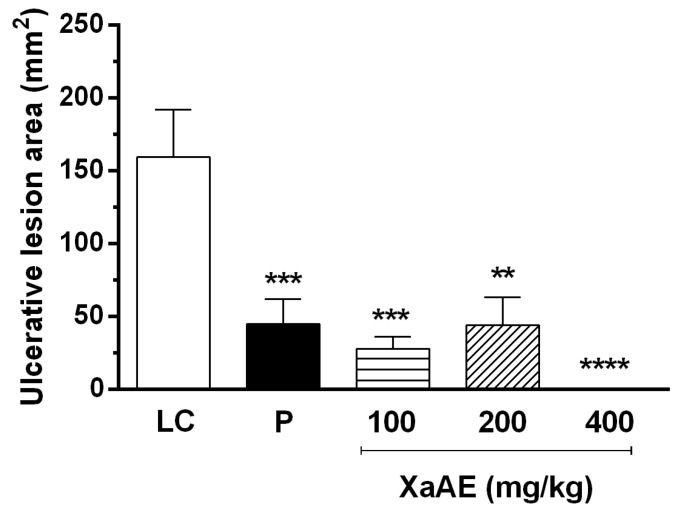
Gastroprotective effect of *X. americana* (XaAE) aqueous extract on absolute ethanol-induced (99%, 1 mL, orally) gastric lesions in Wistar rats. Animals received 0.9% NaCl solution (lesioned control, LC, 10 mL/kg, orally), pantoprazole (P, 40 mg/kg, orally) or XaAE (100, 200 and 400 mg/kg, orally). Results were expressed as mean ± SEM (6–7 animals/group). Statistically different when compared to the lesioned control group (analysis of variance—ANOVA, followed by Dunnett’s multiple comparisons test, ** *p* < 0.001, *** *p* < 0.0005, **** *p* < 0.0001).

**Figure 4:**
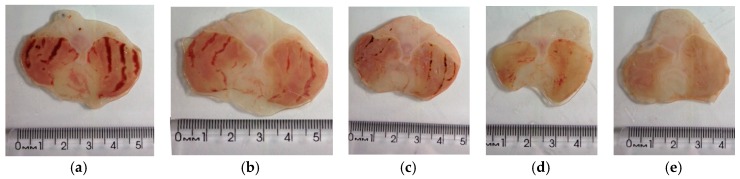
Typical photograph of the gastroprotective effect of aqueous extract of *X. americana* (XaAE) stem bark on absolute ethanol-induced (99%, 1 mL, orally) gastric lesions in rats. (**a**) lesioned control (NaCl 0.9%, 10 mL/kg, orally); (**b**) pantoprazole (40 mg/kg, orally); (**c**–**e**) XaAE (100, 200 and 400 mg/kg, orally, respectively).

Administration of *X. americana* aqueous extract reduced the formation of absolute ethanol-induced ulcerative lesions (Figure 3 and Figure 4). The percentage inhibition of lesions was 76.08%, 77.48% and 100% in rats pre-treated with the extract at doses of 100, 200 and 400 mg/kg, respectively, in contrast with the lesioned control group (LC, 116.5 ± 19.67 mm^2^). Rats treated with pantoprazole showed a reduction of 69.86% of the lesioned area.

#### 2.4.2. Induction of Gastric Lesions by Acidified Ethanol

**Figure 5:**
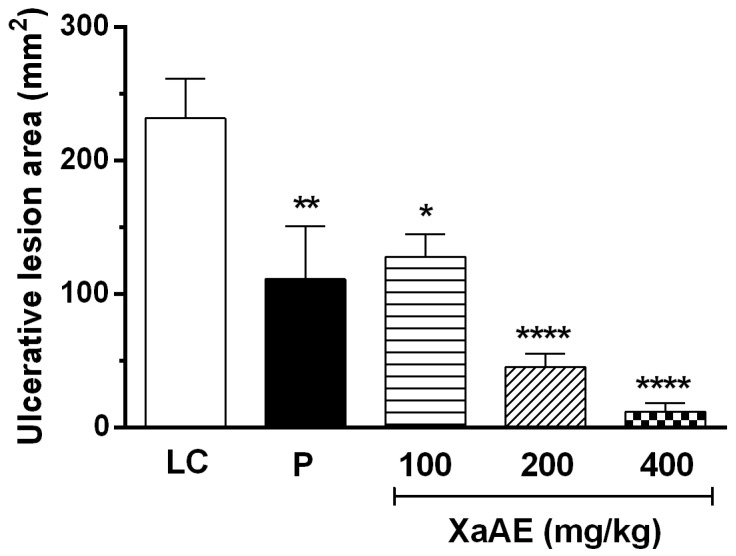
Gastroprotective effect of the aqueous extract of *X. americana (XaAE*) stem bark on acidified ethanol-induced (0.3 M HCl/60% ethanol, 1 mL, orally) gastric lesions in Wistar rats. Animals received 0.9% NaCl solution (lesioned control, LC, 10 mL/kg, orally), pantoprazole (P, 40 mg/kg, orally) or XaAE (100, 200 and 400 mg/kg, orally). Results were expressed as mean ± SEM (6–7 animals/group). Statistically different when compared to the lesioned control group (ANOVA, followed by Dunnett’s multiple comparisons test, * *p* < 0.02, ** *p* < 0.005, **** *p* < 0.0001).

**Figure 6:**
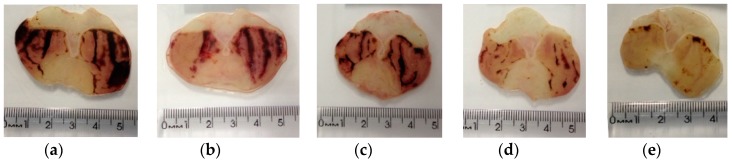
Typical photograph of the gastroprotective effect of aqueous extract of *X. americana* (XaAE) stem bark on ethanol/HCl-induced (0.3 M HCl/60% ethanol, 1 mL, orally) gastric lesions in rats. (**a**) Lesioned control (NaCl 0.9%, 10 mL/kg, orally); (**b**) pantoprazole (40 mg/kg, orally); (**c**–**e**) XaAE (100, 200 and 400 mg/kg, orally, respectively).

*X. americana* aqueous extract promoted significant gastric protection against the acidified ethanol-induced lesions (Figure 5 and Figure 6). The results showed that the rats orally pre-treated with the extract (100, 200 and 400 mg/kg) presented a percentage of inhibition of 44.86%, 80.56% and 94.93%, respectively, in contrast with the lesioned control group (LC, 231.60 ± 29.54 mm^2^). Rats treated with pantoprazole (P) presented a reduction of 51.99% of the lesioned area.

#### 2.4.3. Induction of Gastric Lesions by Indomethacin

**Figure 7:**
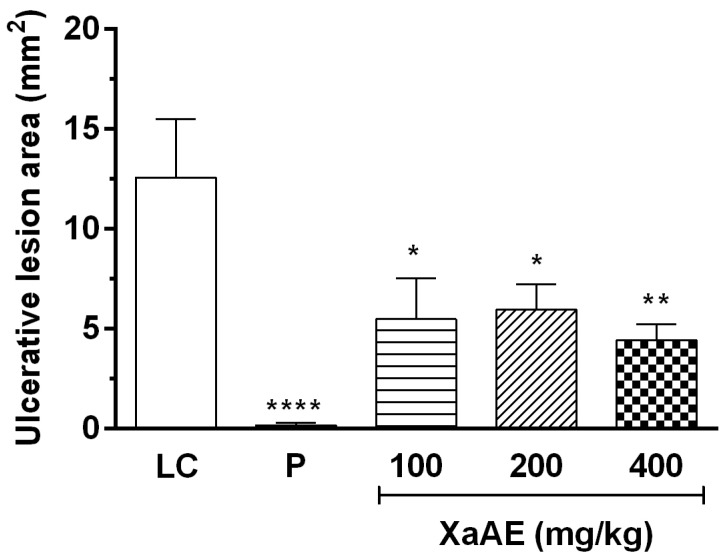
Gastroprotective effect of the aqueous extract of *X. americana (XaAE*) stem bark on indomethacin-induced (30 mg/kg, s.c.) gastric lesions in Wistar rats. Animals received 0.9% NaCl solution (lesioned control, LC, 10 mL/kg, orally), pantoprazole (P, 40 mg/kg, orally) or XaAE (100, 200 and 400 mg/kg, orally). Results were expressed as mean ± SEM (6–8 animals/group). Statistically different when compared to the lesioned control group (ANOVA, followed by Dunnett’s multiple comparisons test, * *p* < 0.05, ** *p* < 0.005, **** *p* < 0.0001).

**Figure 8:**
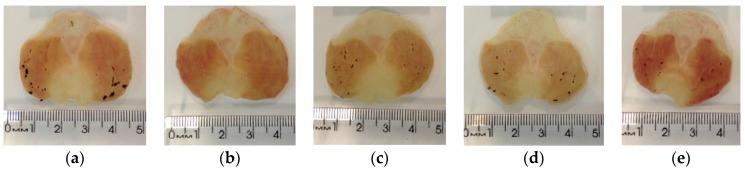
Typical photograph of the gastroprotective effect of aqueous extract of *X. americana* (XaAE) stem bark on indomethacin-induced (30 mg/kg, s.c.) gastric lesions in rats. (**a**) Lesioned control (NaCl 0.9%, 10 mL/kg, orally); (**b**) pantoprazole (40 mg/kg, orally); (**c**–**e**) XaAE (100, 200 and 400 mg/kg, orally, respectively).

*X. americana* aqueous extract promoted significant gastric protection against indomethacin-induced lesions (Figure 7 and Figure 8). The doses of 100, 200 and 400 mg/kg promoted inhibitions of 56.43%, 52.67% and 64.91%, respectively, in contrast with rats of the lesioned control group (LC, 12.56 ± 2.91 mm^2^). Rats treated with pantoprazole (P, 40 mg/kg) showed a reduction of 98.70% of the lesioned area.

#### 2.4.4. Evaluation of Antisecretory Activity

After four hours of pylorus ligation in rats, it was observed that the intraduodenally administered aqueous extract of *X. americana* stem bark (100 mg/kg) reduced the content by 48.65% and the total acidity of the gastric secretion by 32.29% when compared to the lesioned control group (LC, 0.74 ± 0.08 g and 17.90 ± 1.53 Mequiv [H^+^]/mL/4 h, respectively). Ranitidine-treated rats had a 45.95% reduction in secreted gastric contents and 50.34% in total acidity (Table 3).

#### 2.4.5. Evaluation of the Participation of Sulfhydryl Groups (-SH) and Nitric Oxide (ON) in Gastroprotection

**Figure 9:**
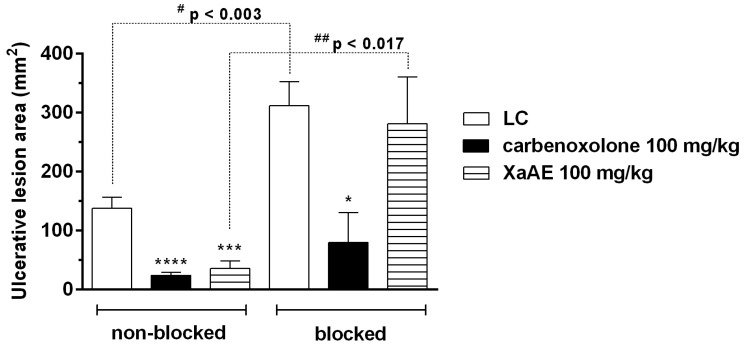
Gastroprotective effect of the *X. americana* aqueous extract (XaAE) stem bark on absolute ethanol-induced (99%, 1 mL, orally) gastric lesions in Wistar rats before (non-blocked, NaCl 0.9%, 10 mL/kg, i.p) and after (blocked) treatment with NEM (10 mg/kg, i.p.). The animals received 0.9% NaCl solution orally (lesioned control, LC, 10 mL/kg), carbenoxolone (orally) or XaAE (orally). Results were expressed as mean ± SEM (6–7 animals/group). Statistically different when compared to the lesioned control group (ANOVA, followed by Dunnett’s multiple comparisons test, * *p* < 0.05, *** *p* < 0.0005, **** *p* < 0.0001). ^#^ The non-blocked and blocked control groups were compared by an unpaired Student’s *t*-test, ^##^ The non-blocked and blocked XaAE 100 mg/kg groups were compared by an unpaired Student’s *t*-test.

As expected, in the absence of blockade, XaAE aqueous extract and carbenoxolone reduced the gastric lesions in rats by 82.69% and 74.17%, respectively, when compared to the lesioned control group (LC, 137.40 ± 18.90 mm²). However, in the presence of blockade, i.e., in the presence of sulfhydryl group inhibitor *N*-ethylmaleimide (NEM, 10 mg/kg, i.p.), the gastroprotective effect of *X. americana* aqueous extract was reversed (9.92%) when compared to the lesioned control group (LC, 311.60 ± 40.82 mm²). In turn, carbenoxolone continued to be active, promoting a significant reduction of 74.48% of the gastric lesions. The comparisons between the groups before and after blockade are also shown in Figure 9.

**Figure 10:**
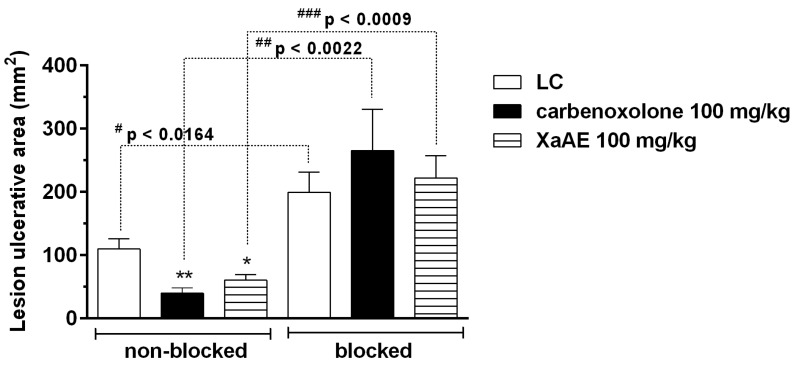
Gastroprotective effect of the *X. americana* aqueous extract (XaAE) stem bark on absolute ethanol-induced (99%, 1 mL, orally) gastric lesions in Wistar rats before (non-blocked, NaCl 0.9%, 10 mL/kg, i.p.) and after (blocked) treatment with L-NAME (70 mg/kg, i.p). The animals received 0.9% NaCl solution orally (lesioned control, LC, 10 mL/kg), carbenoxolone (orally) or XaAE (orally). Results were expressed as mean ± SEM (6–7 animals/group). Statistically different when compared to the lesioned control group (ANOVA, followed by Dunnett’s multiple comparisons test, * *p* < 0.05, ** *p* < 0.005). ^#^ The non-blocked and blocked control groups were compared by an unpaired Student’s *t*-test, ^##^ The non-blocked and blocked carbenoxolone 100 mg/kg groups were compared by an unpaired Student’s *t*-test. ^###^ The non-blocked and blocked XaAE 100 mg/kg groups were compared by an unpaired Student’s *t*-test.

Administrations of *X. americana* aqueous extract and carbenoxolone reduced the gastric lesions area in rats by 64.00% and 45.10%, respectively, when compared to the lesioned control group (LC, 109.43 ± 16.08 mm²) in the non-blocked condition. However, after blockade with the nitric oxide synthase inhibitor *N*ω-nitro-l-arginine-methyl-ester (L-NAME, 70 mg/kg, i.p.), there was no inhibitory effect of aqueous extract of *X. americana* and carbenoxolone on the gastric lesions in relation to the lesioned control group (199.20 ± 31.86 mm²). The comparisons between the groups before and after blockade are also shown in Figure 10. Depletion of sulfhydryl and nitric oxide groups by pre-treatment with NEM and L-NAME, respectively, were able to eliminate the gastroprotective effect of XaAE.

#### 2.4.6. Determination of Mucus Concentration of the Gastric Mucosa

Ligation of the pylorus in the lesioned control group rats promoted a significant decrease in gastric mucus levels (7.89 ± 0.56 μg Alcian blue/g of tissue) compared to those of the false-operated group (non-lesioned, 13.35 ± 0.96 μg of Alcian blue/g of tissue).

**Figure 11:**
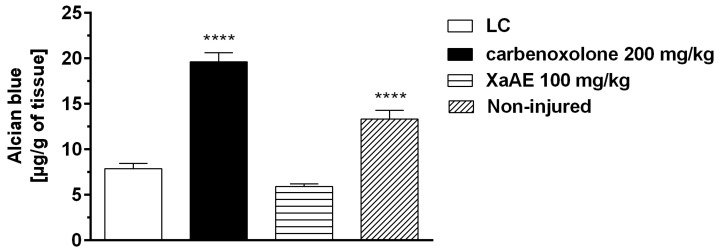
Effect of the aqueous extract from *X. americana* (XaAE) stem bark on the gastric mucus content after pylorus ligation in Wistar rats. The animals received 0.9% NaCl solution orally (lesioned control, LC, 10 mL/kg), carbenoxolone (orally) or XaAE (orally). The non-lesioned group received no treatment. Results were expressed as mean ± SEM (6–7 animals/group). Statistically different when compared to the lesioned control group (ANOVA, followed by Dunnett’s multiple comparisons test, **** *p* < 0.0001).

Rats treated with XaAE did not have an increased of gastric mucus levels (5.93 ± 0.26 μg Alcian blue/g of tissue) compared to the lesioned control group (LC, 7.89 ± 0.56 μg Alcian blue/g of tissue). However, carbenoxolone (CBX, 200 mg/kg) promoted a significant increase in gastric mucus levels of 148.79% in relation to the lesioned control group (Figure 11).

## 3. Discussion

This study investigated the antiulcerogenic pharmacological activity of the aqueous extract of *X. americana* stem bark in acute gastric lesions induced by ethanol, acidified ethanol (HCl/ethanol) and indomethacin, as well as its influence on gastric acid secretion parameters, sulfhydryl groups, nitric oxide, and mucus.

The results obtained demonstrate for the first time that the aqueous extract of the *X. americana* has a gastroprotective effect in the acute models tested. These models are the most used because they represent the etiological agents most commonly involved in gastric ulcers [19]. Hydrochloric acid, free radicals, ethanol and non-steroidal anti-inflammatory drugs (NSAIDs) and *H. pylori* are some of the aggressive factors [4,20,21].

In the acute toxicity test, *X. americana* aqueous extract, when given orally at the dose of 2000 mg/kg, did not produce signs of toxicity or death. These data indicate that its LD_50_ is above 2000 mg/kg and, therefore, can be considered non-toxic under the present experimental conditions. Although Maikai et al. [22] did not register death loss (DL_50_ > 5000 mg/kg) in mice and rats by oral and intraperitoneal administration of the aqueous extract of *X. americana* stem bark, clinical signs of toxicity such as excitement, restlessness, difficulty breathing, loss of appetite, general weakness and depression were observed at high doses, as well as body mass loss. Similar results to ours were obtained by Agyigra et al. [23] who estimated a DL_50_ by oral administration of the methanolic extract of *X. americana* stem bark of more than 5000 mg/kg in rats and mice.

Ethanol is considered to be one of the most intense agents inducing gastric lesions because it promotes serious mucosal disorders due to its direct action, while gastric acidity has little effect on the formation of ulcers [24,25].

Lesions formed by ethanol result from a series of factors such as decreased gastric mucus production, prostaglandins, and sulfhydryl groups, decreased gastric motility, altered transmucosal potential and gastric mucosal blood flow, as the increased generation of free radicals, the release of histamine, ischemia and gastric vascular permeability [20]. Its administration produces hemorrhagic, and necrotic lesions in the mucosa consisting of elongated bands generally parallel to the longitudinal axis of the stomach [26]. The presence of HCl accelerates the process [24]. Oral administration of XaAE reduced the ethanol- and HCl/ethanol-induced gastric lesions at all doses tested, indicating a gastroprotective action.

Indomethacin has been reported to decrease antioxidant enzyme activity [27], increase oxidative stress and inhibit cyclooxygenase (COX), inducing gastric ulceration in the stomach [28,29]. This ulcerogenic mechanism of indomethacin is attributed to a reduced secretion of bicarbonate, mucus and blood flow and inhibition of mucosal repair [30].

In the indomethacin-induced acute ulcer model, in which prostaglandins are suppressed, the extract was able to reduce gastric lesions initially suggesting an inhibitory action on the cyclooxygenase pathway, reducing the production of cytoprotective prostaglandins. However, other mechanisms may contribute to this activity, since Soro [12] showed that the aqueous extract of the *X. americana* stem bark (25, 50 and 100 mg/kg, i.p.) presented analgesic activity in abdominal writhing tests by acetic acid and formalin only in the 2nd phase, which characterizes the possible anti-inflammatory action of the extract.

Proton pump inhibitors are “prodrugs” considered to be very effective and relatively equivalent, reducing by up to 95% the daily production of gastric acid [31]. Pantoprazole has a linear (and therefore more predictable) pharmacokinetic profile, less variable bioavailability and a rapid onset of action when compared to other class representatives such as omeprazole and lansoprazole [32]. In all models of induction of acute gastric lesions, rats treated with pantoprazole showed a reduction of the lesioned area.

The phytochemical study determined the presence of alkaloids, flavonoids, lignans, monoterpenes, sesquiterpenes and diterpenes, naphthoquinones, saponins, hydrolysable tannins, triterpenes, and steroids. The HPLC-DAD-MS/MS identified procyanidins B, procyanidin C, and catechin/epicatechin as the most abundant compounds.

At present, there is no way to attribute the gastroprotective activity to one of the identified major compounds and consequently to estimate their potency. We intend to try to correlate the effect of the extract with those obtained from the isolated compounds. However, the possibility of synergistic interactions between these compounds in the crude extract may make it difficult to establish some correlation.

Shettar [13] reported the presence of flavonoids in phytochemical analysis and their high antioxidant potential of the aqueous extract of *X. americana* leaves. His studies suggest that leaves can be used as natural antioxidants to prevent the incidence and progression of many diseases. In recent years, flavonoids have been the most widely studied natural compounds with potential for antiulcer activity [33,34,35]. In this group, the catechins are the ones with the most well-defined mechanism of action, which include histidine decarboxylase inhibition [19,36] and proton pump inhibition [37].

The gastroprotective activity for epicatechin was also reported by Rozza et al. [38] that demonstrated that this protection of mucosa occurs by stimulation of the increase of the mucus barrier and neutralizing the gastric juice, as well as through the involvement of SH compounds, adrenergic activation, NO, SOD and HSP-70. Catechin and its derivatives act as gastroprotective agents by inhibition of gastric H^+^,K^+^-ATPase [37]. Catechin also demonstrated a gastroprotective mechanism of endocrine action by reducing levels of gastrin and histamine [39]. Lewis [40] also was shown the gastroprotective activity of proanthocyanidin B-2 in mice against stress-induced gastric lesions.

The obstruction promoted by the pyloric ligature causes accumulation of acid in the gastric content and exaggerated distension of the mucosa, consequently leading to gastric hypersecretion and the formation of lesions [41]. In addition, the formation of endogenous histamine and its release from mast cells also is related to the pathogenesis of gastric ulcers produced by pyloric ligation, suggesting that H_2_ antihistamines and histidine decarboxylase inhibitors may be useful in the prevention of such lesions [36]. In this model, treatment with the aqueous extract of *X. americana* stem bark reduced both the volume and total acidity of gastric juice, the major parameters of basal gastric secretion. Considering that inhibition of the proton pump is one of the mechanisms of action associated with catechins [37], it is possible to suggest that the inhibitory action of gastric acidity of XaAE may be related to the presence of these constituents.

In order to understand the mechanisms involved in the gastroprotective action promoted by the XaAE, experimental models have been performed in order to investigate the participation of sulfhydryl groups (-SH), nitric oxide and mucus.

Endogenous sulfhydryl compounds are important for maintaining the integrity of the gastric mucosa, by participating in the production of mucus and binding to free radicals formed during inflammation or exposure to harmful agents, promoting neutralization [42,43]. Ethanol decreases the activity of -SH groups, causing oxidative stress, and confirming the involvement of these groups with the gastroprotective effect [43]. According to El-Ashmawy et al. [44] the garlic extract, which is rich in sulfhydryl compounds, demonstrated antioxidant and anti-inflammatory activity required for protection of gastric mucosa.

Changes in intracellular redox status including the increase in intracellular levels of reactive oxygen species, such as superoxide anion (O_2_^−^), hydroxyl radical (OH) and hydrogen peroxide (H_2_O_2_), decreased levels of sulfhydryl compounds and mainly peroxidation, are important markers of oxidative stress involved in the pathogenesis of various inflammatory or tumoral diseases [45,46]. However, multiple studies suggest the protective effect of medicinal plants and extracts in the treatment and prevention of the burden of tissue oxidative stress, with a significant improvement in lipid peroxidation biomarker levels [47,48,49].

In this study, data demonstrated that gastric exposure to ethanol-associated to depletion of the sulfhydryl compounds promoted by NEM (-SH group inhibitor) decreased the gastroprotection of mucosa. Our results also showed that treatment with NEM eliminated the gastroprotective effect of the extract previously observed, suggesting a possible participation of sulfhydryl groups in its action.

NO also plays a mucosal defensive role by regulating the blood flow and stimulating gastric mucus secretion by the activation of the enzyme guanylyl cyclase [50]. Inhibition of nitric oxide synthase (NOS) by L-NAME results in a decrease in this endogenous mediator and, consequently, an increase of the gastric lesion [51]. In our experiment, the blockade on NOS by L-NAME promoted a significant increase in gastric lesions and also reversed the gastroprotective action of *X. americana* aqueous extract, also indicating a possible contribution of this mediator. It should be noted that although the 70 mg/kg dose of L-NAME with a focus on gastroprotection mechanisms involving the role of nitric oxide as a mediator of gastroprotection has been obtained from studies in the literature [51,52,53,54], this same dose is also capable of increasing the blood pressure of rats [55].

The primary function of the mucus layer is structural, creating a stable layer that supports surface contact with acid and acting as a protective physical barrier against luminal pepsin. Secretion of bicarbonate (HCO_3_^−^) adhered to the mucus layer (stable and adherent gel) creates a nearly neutral pH gradient on the epithelial surface of the stomach and duodenum, providing the first line of defense against luminal acid [56].

Carbenoxolone, the cytoprotective agent used as the standard control in the model of determination of gastric mucus concentration, is a semi-synthetic derivative of the glycyrrhizinic acid, the active ingredient of *Glycyrrhiza glabra*, which has been extensively studied and reported to have anti-ulcer effects in experimental animals and humans [7]. Carbenoxolone maintains the prostaglandin content of the gastric mucosa at high levels due to its inhibitory action on the catabolic enzymes 15-hydroxy-prostaglandin-dehydrogenase and Δ13-prostaglandin-reductase, besides increasing cyclic AMP levels by inhibition of mucosal phosphodiesterases. High levels of prostaglandins promote the action of mucosal defense against ulceration [40]. Our data show that carbenoxolone increased the concentration of Alcian blue complexed to mucus, but this effect was not verified with the extract of *X. americana*, thus initially rejecting the hypothesis of the contribution of mucus in the gastroprotective effect of the extract.

## 4. Materials and Methods

### 4.1. Collection of Plant Material

*X. americana* L. stem bark was collected in the Letras rach (Coordinates: 08°14′05.69″ S, 039°09′31.39″ W, 425 m altitude), in the municipality of Salgueiro, state of Pernambuco, Brazil, during the flowering period in November 2015. Plenty of material of the sampled species was deposited in the Semi-Arid Tropic Herbarium (HTSA), of Embrapa, in the municipality of Petrolina, state of Pernambuco, Brazil, under registry number HTSA 6633.

### 4.2. Extract Preparation and Phytochemical Profile (TLC)

To prepare the extract, stem barks were powdered in a knife mill (Wyllie Macro—TE 650), and then immersed in distilled water, using the proportion of 20% of their dry weight, and reserved for 24 h. The solution was then filtered and lyophilized. The yield of *X. americana* L. (XaAE) stem bark aqueous extract was 15.76% (*w*/*w*), which was stored under refrigeration (6–10 °C) until pharmacological tests and phytochemical analyses. The extract was previously analyzed by thin layer chromatography (TLC) to identify the phytochemical classes [57].

### 4.3. High Performance Liquid Chromatography (HPLC-DAD-MS/MS)

The extract was subjected to Liquid Chromatography coupled to Mass Spectrometry (LC-MS), using an octadecylsilane column (250 × 4.6 mm, 5 μm, Luna C_18_, Phenomenex-Shimadzu, Kyoto, Japan) as stationary phase, and the mobile phase consisted of 2 solvents: solvent A-0.1% formic acid in ultrapure water and solvent B-0.1% formic acid in methanol (HPLC grade) with flow rate of 1.0 mL/min.

The stationary phase was maintained at 30 °C and the volume injected was 20 μL for the samples (1 mg/mL) in the high performance liquid chromatography coupled to the diode array detector and the mass spectrometer (HPLC-DAD-MS/MS) for monitored analyses of 190 to 400 nm and 50 to 1000 *m*/*z*. The analyses used an LC-20 (Shimadzu, Kyoto, Japan) equipped with a quaternary pump system model LC-20ADVP, degasser model DGU-20A, PDA detector model SPD-20AVP, oven model CTO-20ASVP, automatic injector model SIL-20ADVP and controller model SCL-20AVP coupled to a mass spectrometer from AmaZonL (Bruker Daltonics, Billerica, MA, USA) equipped with an electrospray ionization source operating in the analyzer mode and by capture of positive and negative ions to divide the HPLC eluent and a flow rate of 0.2 mL/min was introduced at the source. The following parameters of the mass spectrometer were used: capillary voltage of 3.5 kV; desolvation temperature of 320 °C; gas flow of 10 L/min; and pressure of 60 psi, using nitrogen as drying and nebulization gas.

### 4.4. Animals

Three-month-old Wistar rats (*Rattus norvegicus*) and Swiss mice (*Mus musculus*) of both sexes were provided by the Vivarium of the Department of Physiology and Pharmacology of the Federal University of Pernambuco (DFF/UFPE) and the Aggeu Magalhães/Fiocruz Research Center (CPqAM/Fiocruz), respectively. The animals were kept under controlled lighting, temperature (22 ± 3 °C) and humidity (55–60%) conditions, and received standard water and diet *ad libitum*. The experimental protocols were submitted and approved by the UFPE Ethics Committee on Animal Use (CEUA) of the UFPE (Process 0021/2016).

### 4.5. Acute Toxicity

Acute toxicity was evaluated in Swiss mice of both sexes (*n* = 3/group) [58]. The animals were deprived of food for 12 h and then given a single oral dose of XaAE (2000 mg/kg) or vehicle (0.9% NaCl solution, 10 mL/kg). After administration, the animals were observed individually during 30, 60, 120, 180, 240, 300 and 360 min intervals and, daily for 14 days. The following parameters were evaluated: behavioral changes, clinical signs of toxicity, consumption of water and feed (non-cumulative), and variation of body mass [59].

### 4.6. Experimental Protocols

#### 4.6.1. Induction of Gastric Lesions by Absolute Ethanol

After 16 h of fasting, the animals (*n* = 6–7/group) were pre-treated orally with 0.9% NaCl (lesioned control, LC), pantoprazole (P, 40 mg/kg) or XaAE (100, 200 and 400 mg/kg) one hour before administration of the ulcerogenic agent (absolute ethanol, 1 mL, orally) according to the method described [60], with minor modifications. The animals were euthanized in a CO_2_ chamber one hour after the induction of the lesions. The stomachs were removed, photographed and the gastric ulcer area was determined with the aid of Image J Software version 1.4 (Bethesda, MD, USA). The results were expressed as ulcerative lesion area (mm^2^).

#### 4.6.2. Induction of Gastric Lesions by Acidified Ethanol (HCl/Ethanol)

After 16 h of fasting, the animals (*n* = 6–7/group) were pre-treated orally with 0.9% NaCl solution (lesioned control, LC), pantoprazole (P, 40 mg/kg) or XaAE (100, 200 and 400 mg/kg) one hour before administration of the ulcerogenic agent (solution 0.3 M HCl/60% ethanol, 1 mL orally) according to the method described [61], with minor modifications. The animals were euthanized in a CO_2_ chamber and the gastric ulcer areas were determined as previously described.

#### 4.6.3. Induction of Gastric Lesions by Indomethacin

After 16 h of fasting, the animals (*n* = 6–8/group) were pre-treated orally with 0.9% NaCl (lesioned control, LC), pantoprazole (P, 40 mg/kg) or XaAE (100, 200 and 400 mg/kg). After 30 min of treatment, gastric lesions were induced with indomethacin (30 mg/kg, s.c.) according to the method described [62], with minor modifications. The animals were euthanized in a CO_2_ chamber 6 h after the induction of the lesions and the gastric ulcer areas were determined as previously described.

#### 4.6.4. Evaluation of Antisecretory Activity

This methodology was performed according to the protocol previously described [63], with some adaptations. The animals were divided into 4 groups (*n* = 5–7) and fasted for 16 h with free access to 5% glycoside water solution. For pylorus ligation, the animals were anesthetized (xylazine 6 mg/kg associated with ketamine 60 mg/kg, i.p.), the abdomen was opened for exposure of the stomach and the pylorus was tied with a suture. Immediately after pylorus ligation, NaCl solution 0.9% (lesioned control), ranitidine (60 mg/kg) or XaAE (100 mg/kg) were administered intraduodenally and the abdominal wall was sutured. Four hours later, the animals were euthanized by CO_2_ asphyxiation, the esophagus was pinched, the stomach removed, and the gastric contents collected and centrifuged at 176× *g*/30 min. Weight and pH of the gastric contents were determined. Total acidity of the gastric contents was determined by titration, using 1% phenolphthalein as indicator. The total concentration of the acid was expressed as Mequiv. [H^+^]/mL/4 h. For this experiment, a non-lesioned control group was used, in which the animals did not receive intraduodenal treatment, but underwent surgery stress.

#### 4.6.5. Evaluation of the Participation of Sulfhydryl Groups (-SH) and Nitric oxide (NO) in Gastroprotection

For determination of the role of sulfhydryl groups and nitric oxide, after 24 h of fasting the animals were separated into 12 groups (*n* = 6–7). Three groups received pre-treatment with 0.9% NaCl (lesioned control, i.p.) and three groups with *N*-ethylmaleimide (NEM, 10 mg/kg, i.p.). To evaluate the participation of NO, three groups were pre-treated with 0.9% NaCl (lesioned control, i.p.) and three groups treated with *N*ω-nitro-l-arginine-methyl-ester (L-NAME, 70 mg/kg, i.p.). After 30 min of pre-treatment, each group received an oral dose of NaCl 0.9% carbenoxolone (CBX, 100 mg/kg) and XaAE (100 mg/kg). After 1 h, all the animals received 1 mL of absolute ethanol (orally) for induction of gastric lesions. After 1 h of ethanol administration, the rats were euthanized in a CO_2_ chamber, the stomachs removed and opened along the greater curvature. The stomachs were removed, photographed and the gastric lesion area was determined with the aid of Image J Software. The results were expressed as ulcerative lesion area (mm^2^) [51,64].

#### 4.6.6. Determination of Mucus Concentration of the Gastric Mucosa

The content of soluble glycosaminoglycans in the gastric mucosa was investigated using the Alcian blue dye, specific for acid mucins. The animals were divided into four groups (*n* = 6–7): NaCl 0.9% (lesioned control), carbenoxolone (CBX, 200 mg/kg), XaAE (100 mg/kg) and Sham-operated (non-lesioned control). After 1 h of treatment, the animals were anesthetized (xylazine, 6 mg/kg associated with ketamine, 60 mg/kg, i.p.) and the pylorus was tied. After 4 h of ligation, the animals were euthanized, and the esophagus pinched so that stomach contents were not lost. The stomachs were removed, washed out with distilled water, dried, and opened along the great curvature to remove the gastric material to be weighed. The non-glandular portion was discarded, and the glandular portion was weighed and reserved in an individual container with 10 mL of 0.1% Alcian blue solution for 2 h. Excess of dye was removed by washing the stomach with 7 mL of 0.25 mol/L sucrose solution; a first wash was performed for 15 min and a second for 45 min. The dye complexed to the mucus of the stomach wall and it was removed with 10 mL of 0.5 mol/L magnesium chloride solution, mixing by vortexing, for 1 min in intervals of 30 min, for 2 h. The stomachs were removed and Falcon tubes with the solution were stored in the refrigerator for approximately 8 h. After this time, the tubes were shaken for 1 min, aliquots of 4 mL were collected and added to 4 mL of ethyl ether and vortexed for 2 min. The obtained emulsion was centrifuged for 10 min at 1016.6× *g*/25 °C. The tubes were placed in an ice bath, the film formed above the supernatant was discarded and the aqueous phase separated. The results were obtained by spectrophotometric reading at 595 nm and interpolated in a standard curve of Alcian blue (100, 50, 25, 12.5 and 6.25 μg/mL), to calculate the concentration (μg) of the dye retained in the stomach in relation to the control group [65].

#### 4.6.7. Statistical Analysis

Values are expressed as mean ± standard error of the mean (SEM). Differences between groups were determined by analysis of variance (ANOVA) followed by Dunnett’s multiple comparison test. The level of significance for rejection of the null hypothesis was 5% (*p* < 0.05). In acute toxicity and influence of sulfhydryl groups and nitric oxide on gastroprotection, an unpaired Student’s t-test was used to check statistical differences between the two groups. Statistical analyses were performed using the GraphPad Prism version 7.0 (GraphPad Software, Inc., La Jolla, CA, USA).

## 5. Conclusions

The set of results indicates that *X. americana* aqueous extract has a gastroprotective action whose initial mechanism of action involves the participation of sulfhydryl groups, nitric oxide, and antisecretory activity. The data also indicate that flavonoids, procyanidins B, procyanidins C, and catechin/epicatechin, identified as major constituents, are related to this activity.

## Figures and Tables

**Table 1:** Metabolic compounds identified in the phytochemical characterization of *X. americana* (XaAE) aqueous extract.

Classes of Secondary Metabolites	Standards	Revealing Agent	Intensity
Alkaloids	Yohimbine	Dragendorff	+
Flavonoids	Quercetin	NP/PEG	+++
Lignans	-	Vanillin phosphoric	+++
Mono, sesqui and diterpenes	-	Vanillin sulphuric	+++
Naphthoquinones	-	10% Ethanolic KOH	+++
Saponins	Saponin	Sulfuric vanillin	+
Hydrolysable tannins	Gallic acid	Ferric chloride 2%	++
Triterpenes and steroids	-	Sulfuric vanillin	+++

Natural products-polyethylene glycol reagent (NP/PEG) (=NEU-reagent), KOH: potassium hydroxide. (+++) strong; (++) medium; (+) weak.

**Table 2:** Compounds detected in *X. americana* aqueous extract.

Retention Time (min)	*m*/*z* (+)	MS^2^ (+)	*m*/*z* (−)	MS^2^ (−)	λ Max (nm)
7.8	355.11	343.87	168.91	124.91	278
16.8	579.14	427.11/291.07	577.16	425.04/288.95	278
18.9	867.20	-	866.19	695.12/577.16/407.04	278
20.6	603.10	313.02	289.03	245.02/204.96	278
32.3	459.11	415.13/353.05/291.09	435.16	329.03/166.91	278

**Table 3:** Effect of *X. americana* (XaAE) aqueous extract on gastric secretion parameters in Wistar rats subjected to pylorus ligation.

Groups	Dose (mg/kg, i.d.)	Gastric Content (g)	Gastric pH Value	Total Acidity (Mequiv. [H^+^]/mL/4 h)
Non-lesioned control	-	0.23 ± 0.03 ****	4.39 ± 0.51 *	12.13 ± 2.16 *
LC	-	0.74 ± 0.08	3.25 ± 0.10	17.90 ± 1.53
Ranitidine	60	0.40 ± 0.08 **	3.83 ± 0.15	8.89 ± 0.99 ***
XaAE	100	0.38 ± 0.07 **	3.40 ± 0.08	12.12 ± 0.84 *

LC: lesioned control group (NaCl 0.9%, 2.5 mL/kg, intraduodenally). Values represent mean ± SEM (5–7 animals/group). Statistically different when compared to the lesioned control group (ANOVA, followed by Dunnett’s multiple comparisons test, * *p* < 0.05, ** *p* < 0.005, *** *p* < 0.0005, **** *p* < 0.0001).
